# NRF2 Mediates Therapeutic Resistance to Chemoradiation in Colorectal Cancer through a Metabolic Switch

**DOI:** 10.3390/antiox10091380

**Published:** 2021-08-28

**Authors:** Séan M. O’Cathail, Chieh-Hsi Wu, Rachael Thomas, Maria A. Hawkins, Tim S. Maughan, Annabelle Lewis

**Affiliations:** 1 Institute of Cancer Sciences, College of Medical & Veterinary Life Sciences, University of Glasgow, Glasgow G12 8QQ, UK; 2 Mathematical Sciences, University of Southampton, Southampton SO17 1BJ, UK; c-h.wu@soton.ac.uk; 3 Wellcome Trust Centre for Human Genetics, University of Oxford, Oxford OX1 2JD, UK; rachaelt@bu.edu; 4 Medical Physics and Biomedical Engineering, University College London and University College London Hospitals NHS Foundation Trust, London WC1E 6BT, UK; m.hawkins@ucl.ac.uk; 5 Oxford Institute of Radiation Oncology, University of Oxford, Oxford OX1 2JD, UK; tim.maughan@oncology.ox.ac.uk; 6 Department of Life science, CHMLS, Brunel University London, London UB8 3PH, UK; annabelle.lewis@brunel.ac.uk

**Keywords:** NRF2, radiation, metabolism, therapeutic resistance

## Abstract

Radiation resistance is a significant clinical problem in rectal cancer treatment, the mechanisms of which are poorly understood. NRF2 signalling is known to contribute to chemo/radioresistance in some cancers, but its role in therapeutic resistance in colorectal cancer (CRC) is unexplored. Using siRNA and CRiSPR/Cas9 isogenic CRC cell lines, we investigated the effect of the knockdown and upregulation of the NRF2 pathway on chemo-radiosensitivity. Poly (A) enriched RNA sequencing and geneset enrichment analysis (GSEA) were carried out on both sensitive and resistant cell models for mechanistic insights. Finally, a cohort of rectal patient samples was profiled to understand the clinical relevance of NRF2 signalling. Radioresistant cell lines were significantly radiosensitised by siRNA knockdown (SW1463, SER10 1.22, ANOVA *p* < 0.0001; HT55, SER10 1.17, ANOVA *p* < 0.01), but not the (already) radiosensitive HCT116. The constitutive activation of NRF2 via a CRISPR Cas9 NFE2L2 mutation, E79K, induced radioresistance in HCT116 (SER10 0.71, ANOVA, *p* < 0.0001). GSEA demonstrated significant opposing metabolic dependencies in NRF2 signalling, specifically, the downregulation of amino acid and protein synthesis with low levels of NRF2 and upregulation with over expression. In a clinical cohort of 127 rectal patients, using a validated mRNA signature, higher baseline NRF2 signalling was associated with incomplete responses to radiation higher final neoadjuvant rectal (NAR) score (OR 1.34, 95% C.I. 1.01–1.80, LRT *p*-value = 0.023), where high NAR indicates poor radiation response and poor long-term prognosis. This is the first demonstration of NRF2-mediated radiation resistance in colorectal cancer. NRF2 appears to regulate crucial metabolic pathways, which could be exploited for therapeutic interventions.

## 1. Introduction

Chemoradiation is the standard of care for all patients with locally advanced rectal cancer. Some patients respond poorly with little or no tumour regression [1], highlighting the need to better understand contributing factors. NRF2 is a potent transcription factor that plays a responsive role in cell protection against oxidative and electrophilic stress, as well as metabolic regulation. In unstressed conditions, NRF2 is targeted for ubiquitination by KEAP1 resulting in its degradation. The constitutive activation of NRF2 signalling can occur by a number of distinct mechanisms, including somatic mutation of key regulators KEAP1/NFE2L2, methylation of KEAP1 and direct activation by oncogenes KRAS, BRAF and c-MYC [2,3,4]. This allows NRF2 to function in an ‘oncogene’ fashion, promoting survival, resisting radiation and dysregulating metabolism [5,6]. In fact, NRF2 has been linked, either directly or indirectly, to all of the Hallmarks of Cancer [7].

NRF2 pathway expression has been shown to contribute to radioresistance in many tumour types, in particular, lung cancer [8,9], SCC head and neck [10,11], oesophageal [12] and pancreas [13], and its mechanism extensively investigated [14]. However, its role in therapeutic resistance in CRC is poorly understood, in spite of the fact that the NRF2 pathway can be overexpressed in CRC through a variety of the outlined mechanisms [15]. This oversight is probably due to the fact that the rate of somatic mutation in both KEAP1 and NFE2L2 is low in CRC [16] in comparison to the previously studied tumour types. Nevertheless, in a PanCancer analysis, the level of NRF2 pathway signalling is higher than would be expected (in multiple tumour types, including colon and rectal tumours) given this low mutation rate, suggesting that activation within tumours occurs, warranting further investigation [17].

In this study, we investigated the effects of downregulating and overactivating NRF2 on both chemo- and radio-resistance in colorectal cancer models in vitro, identified the key cellular pathways involved, and assessed the clinical relevance of NRF2 pathway activation in a large clinical rectal cancer dataset.

## 2. Materials and Methods

### 2.1. Cell Culture

Cell lines, SW146 (rectal adenocarcinoma), HT55 (rectal adenocarcinoma) and HCT116 (colon adenocarcinoma), were obtained from the American Type Culture Collection (ATCC) and the European Collection of Authenticated Cell Cultures (ECACC). All cells were maintained in Dulbecco’s modified Eagles Medium (DMEM) supplemented with 10% FBS and 1% penicillin streptomycin. 

### 2.2. NRF2 Disrupted Cell Line Models

Isogenic cell lines HCT116E79K+ and parental HCT116WT were obtained from Horizon Discovery^®^ (Cambridge Research Park, Cambridge, CB25 9TL, UK). The missense mutation was confirmed by Sanger sequencing and FISH was used to confirm that the *NFE2L2* locus was otherwise intact (see supplementary methods). The upregulation of NRF2 target genes was confirmed by quantitative RT-PCR with Taqman probes (Applied Biosystems, Life Technologies Ltd, Paisley, PA4 9RF, UK).

NFE2L2 siRNAs and non-targeting controls (Ambion^®^, see Supplementary Materials for sequences) were transfected into HCT116 and SW1463 using Lipofectamine 2000 (final concentration 1.8 µM, Invitrogen^®^) and HT55 using INTERFERin-HTS (final concentration 20 nM, VWR). Knockdown of NRF2 and target genes was confirmed by quantitative RT-PCR with Taqman probes (Applied Biosystems).

### 2.3. Assessment of Radio and Chemotherapeutic Resistance

Clonogenic assays were carried out on siRNA transfected cells irradiated at 2, 4 and 6 Gy using a caesium-137 irradiator (Gamma Service GSR D1) and incubated for 14–25 days for colony formation. Fixed colonies stained with 0.5% (*w*/*v*) crystal violet were counted digitally using GELCOUNT™ (Oxford Optronix) software. Statistical analysis and plotting of clonogenic survival curves was performed using the ‘CFAssay’ package in R [18].

Cytotoxic drugs were obtained from Sigma (oxaliplatin), Flurochem (5-flurouracil) and Sellekchem (SN-38). Cell viability to cytotoxic drugs was determined using MTS assay (CellTiter^®^ 96R AQueous Non-Radioactive Cell Proliferation Assay), performed as per the manufacturer’s instruction. Analysis was carried out using the R drc package [19].

### 2.4. RNA Sequencing and Analysis

Poly(A) enriched mRNA libraries were prepared using the Truseq stranded mRNA system and sequenced on a HiSeq4000 (Illumina) according to the manufacturer’s protocols. Differential expression analysis was performed in R with both ‘DESeq2’ [20] and ‘edgeR’ [21] to ensure robustness. Main analysis and plots were generated from edgeR data.

GSEA was carried out against the hallmark gene sets, Reactome pathways and KEGG pathways from the Molecular Signatures Database v6.2 [22,23] using the ‘fgsea’ package [24]. The ranked gene list was compared to the a priori defined gene sets and the curated geneset NFE2L2.v2, for a total of 1000 permutations. An FDR < 0.1 was used to highlight significant pathways of enrichment. RNA sequencing data were deposited to the Gene Expression Omnibus under accession number GSE136011. 

### 2.5. Rectal Cancer Cohort and Statistical Association of NRF2 Signature with NAR Score

Patients’ samples were obtained through the MRC Stratified Colorectal cancer (S:CORT) collaboration and approved by the East of England-Cambridge South Research Ethics Committee (NHS Health Research Authority) (REC reference 15/EE/0241). Following RNA extraction from biopsy specimens, samples were profiled on Xcel microarray as per the manufacturer’s instructions. Raw data were normalised and batch corrected to generate an expression matrix. NRF2 pathway expression was measured using a previously published 36-gene signature [25]. Univariate and multivariate ordinal logistic regression models were built, the null hypothesis being (H0) that it would not provide any explanatory power for NAR scores. The likelihood ratio test (LRT) was employed to test the hypothesis. (Full details available in supplementary methods).

## 3. Results

### 3.1. Changes in NRF2 Signalling Mediate Radiation Response

**Figure 1:**
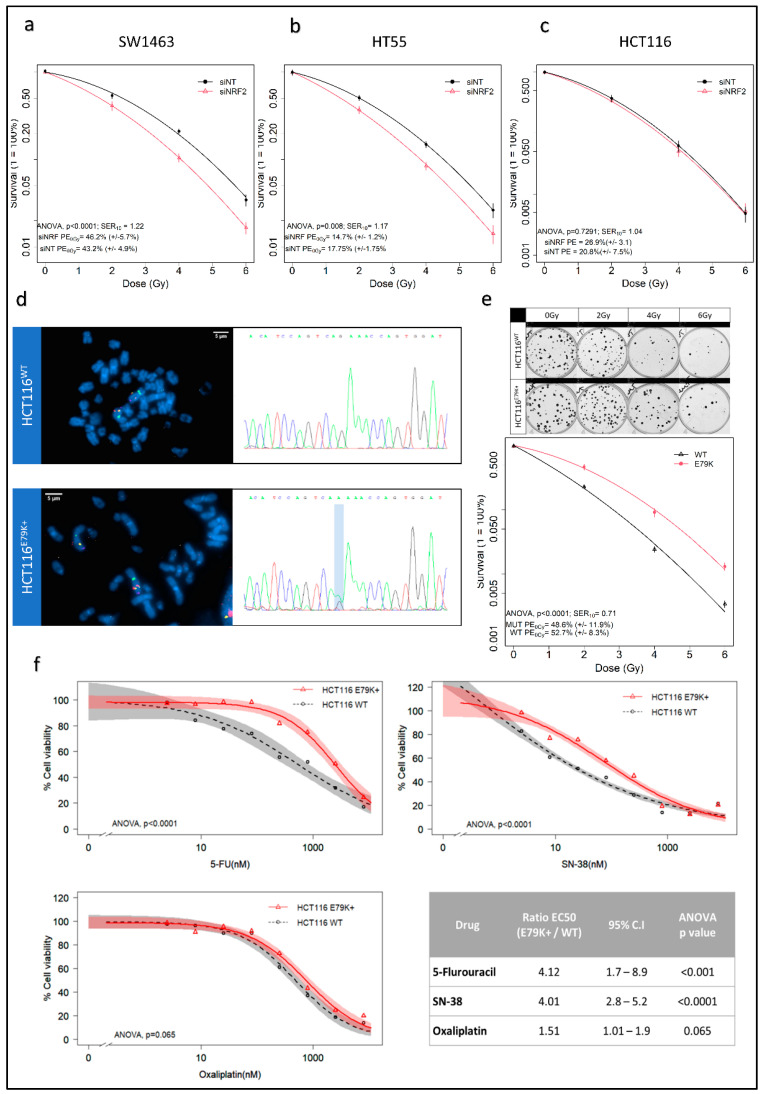
NRF2 knockdown increases radiosensitivity in resistant cell lines and introduction of NRF2 mutation induces radioresistance in sensitive cells. (**a**–**c**) Cell survival curves demonstrating significant radiosensitisation in SW1463 and HT55, but not HCT116; (**d**) FISH analysis of NFE2L2 locus and Sanger sequencing of CRISPR HCT116 isogenic cell lines confirming diploid status of NFE2L2 and point mutation (G > A) at position 235; (**e**) clonogenic assay and resultant cell survival curve demonstrating increased radioresistance in E79K+ mutant cell line relative to wild type; (**f**) MTS cell viability assays with common colorectal chemotherapeutics—5-Flurouracil, Irinotecan, Oxaliplatin—demonstrating increased resistance to both 5-Flurouracil and Irinotecan.

Knockdown of NRF2 with RNAi resulted in a significant decrease in NRF2 signalling in three CRC cell lines as assessed by validated NRF2 transcriptional targets, NQO1 and HMOX1 [26], as surrogates of pathway perturbation [27] (Supplementary Figure S1a–c). Significant radiosensitisation was observed in NRF2-depleted SW1463 (SER10 1.22, ANOVA *p* < 0.0001) and HT55 (SER10 1.17, ANOVA *p* < 0.01) cell lines, with no effect on HCT116 (Figure 1a–c), and was consistent across three independent NRF2 siRNAs (Supplementary Figure S1d). HCT116 is intrinsically a radiosensitive cell line [28]. Compared to SW1463 and HT55, it also had the lowest level of NRF2 activity (Supplementary Figure S1e). This suggested a possible threshold effect for radioresistance, where an a priori degree of overactivation of NRF2 signalling is required for knockdown to produce radiosensitisation. To investigate further, we used HCT116 CRISPR/Cas9 isogenic wild type and NFE2L2 mutant models. Karyotyping, FISH and Sanger sequencing confirmed that both isogenic HCT116 lines were diploid for NFE2L2 and the presence of a single heterozygous missense mutation at position 235 of NFE2L2 in HCT116 E79K+ cells (Figure 1d). The resulting mutant NRF2 protein has weak affinity for KEAP1, resulting in cytoplasmic accumulation (Supplementary Figure S1f) of NRF2. Elevated levels of NRF2 protein and downstream activation were confirmed by mRNA levels of NRF2 transcriptional targets (Supplementary Figure S1g). HCT116 E79K+ demonstrated increased radioresistance compared to wild type (SER10 0.71, ANOVA, *p* < 0.0001) (Figure 1e), confirming that the overactivation of NRF2 signalling induces radioresistance. MTS cell viability assays were performed for 5-FU, SN-38 and Oxaliplatin to identify cross resistance with common radiosensitising chemotherapies. Relative to HCT116WT, HCT116 E79K+ showed significantly increased cell viability to 5-FU and SN-38 but not oxaliplatin (Figure 1f).

### 3.2. NRF2 Signalling Changes Significantly Alter Amino Acid Metabolism

**Figure 2:**
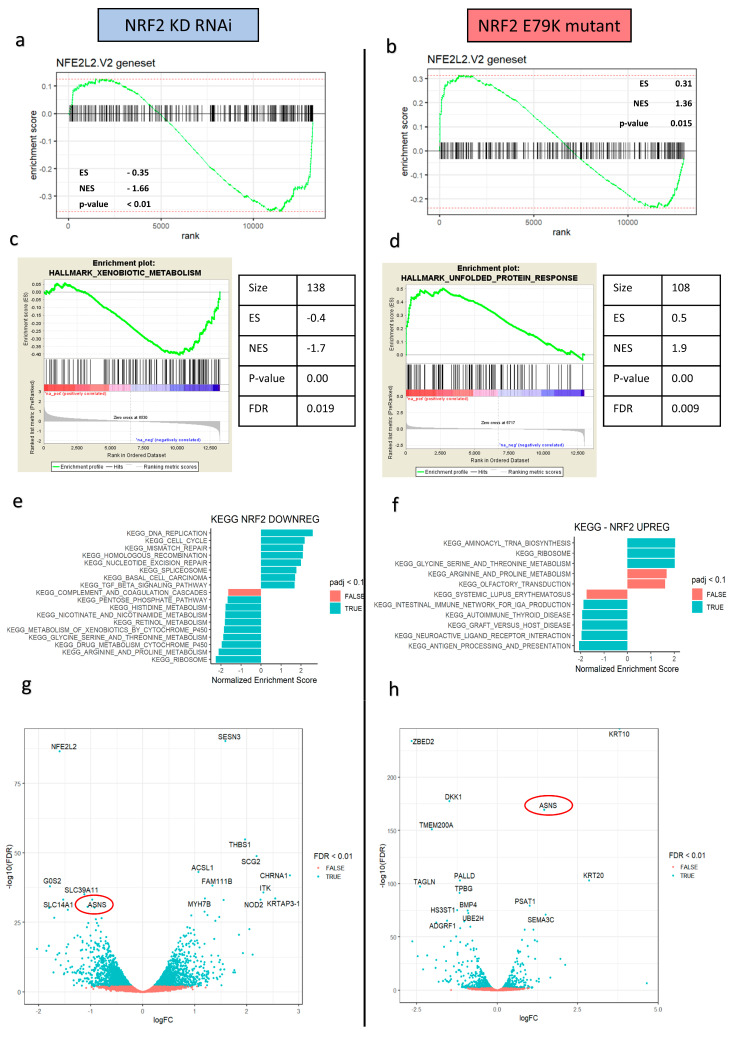
Downregulation and upregulation of NRF2 signalling result in opposing effects on tumour metabolism, particularly amino acid and protein metabolism. (**a**) GSEA enrichment plot for NFE2L2 targets in the ranked logFC of genes in the siRNA SW1463 model showing significant downregulation of NRF2 signalling; (**b**) GSEA enrichment plot for NFE2L2 targets in the ranked logFC of genes in the HCT116 E79K+ model showing significant upregulation of NRF2 signalling; (**c**,**d**) GSEA enrichment plots the most significant Hallmark gene signatures, highlighting an opposing effect on metabolism; (**e**,**f**) KEGG pathway analysis supports the significant opposing effects, with a particular focus on amino acid and protein metabolism; (**g**,**h**) volcano plots highlighting the significant opposing changes in asparagine synthetase (ASNS) expression.

To better understand the NRF2 signalling changes produced in the in vitro colorectal models, which in turn could explain the phenotypic changes in radiation responses, whole transcriptomic profiling RNA sequencing and gene set enrichment analysis (GSEA) were performed on the RNAi and isogenic mutant models. Using the NFE2L2.v2 signature from the molecular signatures database to verify that NRF2 signalling had been altered, the RNAi model showed significant negative enrichment for the NFE2L2 targets (ES-0.35; NES-1.66; *p* < 0.01). By contrast, the NRF2 E79K + mutant model showed significant enrichment for the NFE2L2 targets (ES 0.31; NES 1.36; *p* = 0.015) (Figure 2a,b). Global analysis using the Hallmarks genesets highlighted the significant downregulation of metabolism in the NRF2 KD model and metabolic upregulation with NRF2 overactivation (Figure 2c,d). Further phenotypic exploration undertaken using the Kyoto Encyclopaedia of Genes and Genomes (KEGG) pathways and REACTOME pathways also showed significant changes in metabolic processes, in particular, pathways involved in amino acid synthesis and protein metabolism (Figure 2c,d, Supplementary Tables S1 and S2).

The enriched, opposing effects on amino acid metabolism was of particular interest and prompted further investigation as a potential mechanism. One striking overlap of the volcano plots and the top 20 DE genes under both conditions was the presence of asparagine synthetase (ASNS), a significantly downregulated target in the knockdown model (logFC-0.96, FDR 5.66 × 10–34) and a significantly upregulated target in the NRF2 mutant model (logFC 1.46, FDR 2.72 × 10–170). ASNS encodes an enzyme which catalyses the conversion of aspartate and glutamine, to asparagine and glutamate, respectively, in an ATP-dependent reaction. Recent data also suggest that ASNS acts in conjunction with mTOR signalling to promote protein and nucleotide synthesis and cell proliferation [29]. GSEA analysis using the MSigDB C6 Oncogenic signatures set confirmed mTOR expression was shared and highly enriched in both models, with negative enrichment following NRF2 KD (NES-1.77, FDR *p* value = 0.036) and positive enrichment in NRF2 E79K + MUT cells (NES 1.97, FDR *p* value = 0.006) (Supplementary Figure S2a,b; Supplementary Tables S3 and S4).

**Figure 3:**
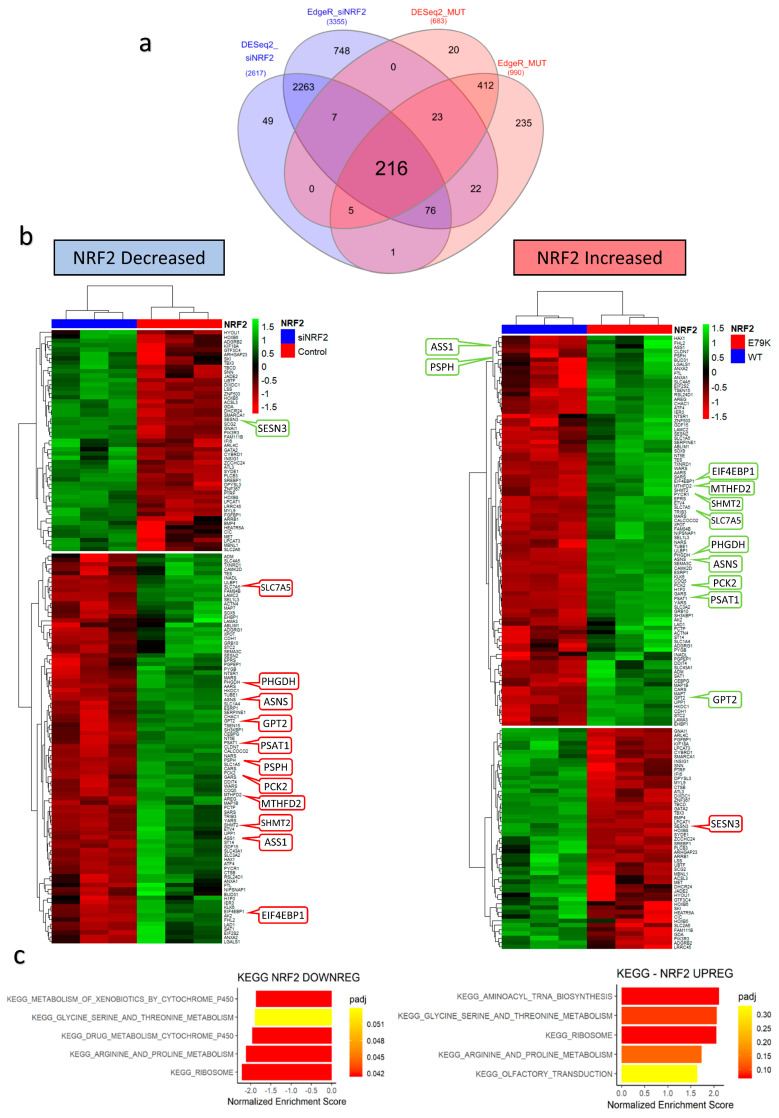
Changes in NRF2 signalling alternatively regulates a conserved group of genes responsible for metabolic processes. (**a**) Venn diagram showing the shared group of genes in different states of NRF2 activation; (**b**) heatmaps of relative change in expression (z-score) of shared genes, with key metabolic genes highlighted; (**c**) GSEA KEGG pathway analysis of conserved group of shared genes demonstrating significant enrichment for amino acid and protein metabolism.

To understand which genes were mediating these changes, all significantly (FDR < 0.01) differentially expressed genes from the NRF2 KD and NRF2 E79K MUT models were examined for overlap. A total of 216 genes were shared, of which 141 had opposing logFC changes (Supplementary Tables S5 and S6) and demonstrated opposing enrichments for metabolic processes (Figure 3). Functional relationship analysis of these genes using the STRING database [30] revealed a highly significant interaction network, with ASNS at the hub (Supplementary Figure S3). The number of interacting nodes was 117, higher than the predicted number of 35 for a set of 141 genes, with a protein–protein interaction enrichment *p*-value < 1 × 10–16. The top biological process (GO) enrichments were GO: 0006521 ‘cellular amino acid metabolic process’ (19/308; FDR *p*-value 7.83 × 10–9) and GO: 0006418 ‘tRNA aminoacylation of protein translation’ (9/48; FDR *p*-value 4.4 × 10–7). The interaction network highlighted three key metabolic domains—amino acid metabolism and transport, tRNA acetylation and mTOR signalling—which correspond to the interactions seen in the KEGG GSEA analysis (Figure 3c).

### 3.3. NRF2 Signalling Associated with Worse Response to Radiotherapy In Vivo

To explore the role of NRF2 signalling in clinical radioresistance, mRNA profiling of a cohort of pre-treatment rectal biopsies specimens was undertaken. NRF2 signalling was derived using a previously validated NRF2 36 gene colorectal mRNA signature (Supplementary Table S7). A total of 127 patients were included with characteristics summarised in Table 1.

Radiation response in patients can be measured in several ways. One of the most common is pathological response, which can be easily binarized into complete (no visible tumour remaining) and incomplete (any visible tumour remaining). A more novel approach is the neoadjuvant rectal (NAR) score [31], which is calculated from clinical variables of tumour (T) and nodal (N) stage. It captures how much a tumour has regressed and whether viable tumour deposits remain in lymph nodes following radiation treatment. NAR is a validated surrogate of the true clinically relevant endpoint of disease-free survival (DFS) and overall survival (OS) [31,32] and is the primary endpoint in the ongoing NRG-GI002 platform phase II trial (NCT02921256).

**Figure 4:**
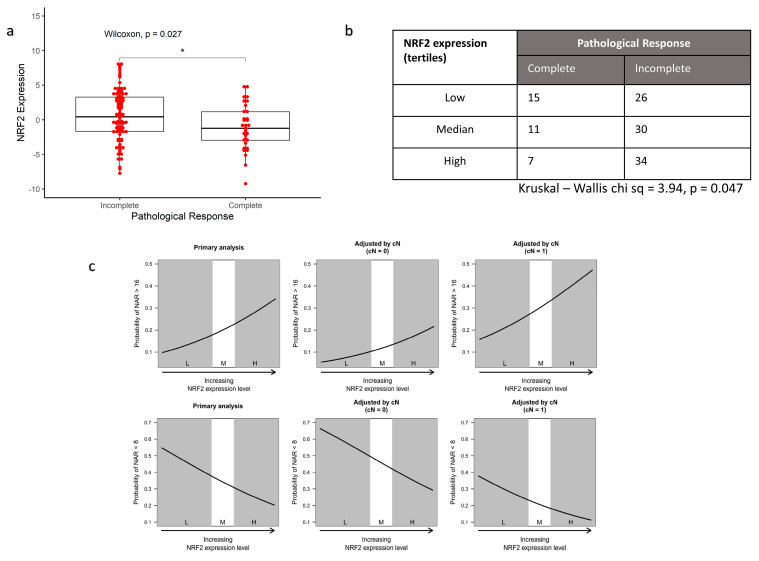
High NRF2 expression in baseline biopsies is associated with worse tumour response in patients (N = 123). (**a**) Boxplot comparing mean NRF2 signature expression between patients who achieve a complete pathological response and those who have residual disease following radiation; (**b**) count table showing a significantly higher proportion of patients with the highest tertile of NRF2 signature expression have a worse response to radiation; (**c**) ordinal logistic regression plots for NRF2 expression predicting NAR score. Upper row: Probability of an NAR > 16. As NRF2 expression increases, the odds of a high NAR score increase. The effects of including clinical nodal stage from the MRI on the model are also shown. Clinical nodal stage is the only clinical factor that is not part of the NAR score. Node positivity at baseline and high expression of NRF2 signature has much greater odds of a high NAR score. Lower row: Probability of NAR < 8. As NRF2 expression increases, the odds of low NAR score decrease. Adjustments for cN stage are also shown. (L, M, H are tertiles of NRF2 signature expression where L = lowest/bottom tertile; M = median tertile; H = highest tertile) (* signifies *p* < 0.05).

Preoperative mean NRF2 expression was significantly higher in those with an incomplete response to radiation (Wilcoxon rank sum, *p* = 0.027) (Figure 4a). The rate of complete response is highest among patients in the lowest tertile of NRF2 expression and lowest in those in the highest tertile (Figure 4b). 

Higher NRF2 signalling was also associated with higher NAR (Figure 4c). In the ordinal regression model, this relationship was statistically significant with an odds ratio of 1.34 (95% C.I. 1.01–1.80, LRT *p*-value = 0.023). After adjusting for the effect of clinical N stage, higher NRF2 signature expression corresponded to higher NAR scores (OR = 1.35, 95% C.I. 1.00–1.81, LRT *p*-value = 0.0403). In clinical terms, the model demonstrates that for patients with high NRF2 activation, as measured by the chosen RNA-based signature, the odds of a high NAR score—a poor response—at the end of neoadjuvant therapy were much increased.

## 4. Discussion

Here, we present the first demonstration that aberrant NRF2 signalling is an important determinant of radiation-induced cell death in colorectal cancer cells. Although previous work has established that the upregulation of NRF2 signalling acts as a mediator of radioresistance in some cancer types [8,11], its effect in CRC had been overlooked and its role underinvestigated. The cell lines SW1463 and HT55, which are known to be radioresistant [28], had higher levels of NRF2 signalling and were significantly sensitised by NRF2 inhibition in a reproducible fashion. Inhibition had no effect on radiosensitive cell lines. However, it was possible to induce radioresistance in the sensitive HCT116 cell line by CRISPR Cas9 constitutive activation of the NRF2 pathway. Previous work has also suggested that increased NRF2 leads to chemoresistance [33,34,35], confirmed by our colorectal drug sensitivity screen. Taken together, these data provide proof of concept that NRF2 may have a role to play in mediating therapeutic resistance in vivo. The idea that baseline, constitutive activation could play a key role in radiation response was explored in a large rectal cancer biopsy cohort. Using a validated RNA-based signature of NRF2 signalling, we further demonstrate that higher baseline levels of NRF2 signalling contribute to a radioresistant phenotype in rectal cancer patients treated with neoadjuvant chemoradiotherapy. These results highlight the real-world clinical relevance of NRF2 in the therapeutic resistance of colorectal cancer.

**Figure 5:**
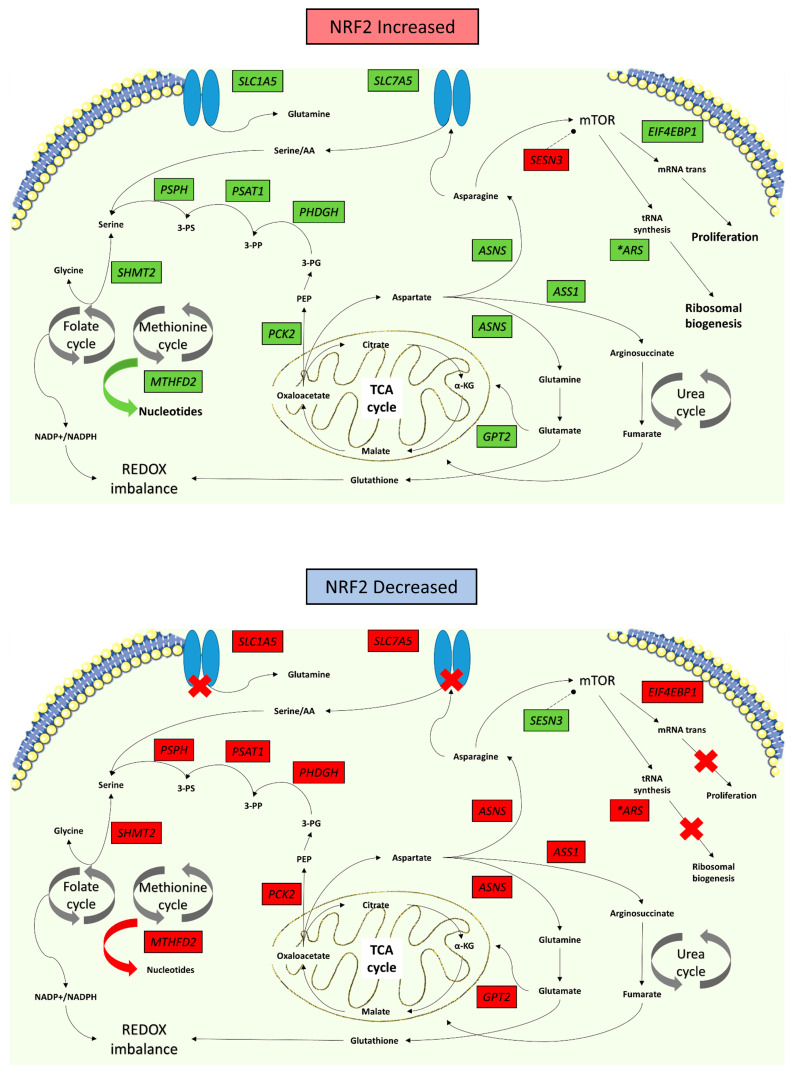
Metabolic ‘switch’ model in response to changes in NRF2. Upregulation of NRF2 facilitates an anabolic process of nucleotide production, mTOR signalling which increases cell proliferation and ribosomal biogenesis. It also impairs the cell’s ability to maintain redox homeostasis. Downregulation of NRF2 produces the reverse effect, abrogating many essential cell mechanisms required for cell survival, crucial to survival following radiation induced damage. The cell circuit diagram summarises the changes produced by the relative change in gene expression (green = upregulated; red = downregulated).

From a mechanistic perspective, these data and the other recent literature [13,36], suggest that NRF2 exerts regulatory control over a complex amino acid metabolic biogenesis program, possibly through co-ordination with mTOR, to increase therapeutic resistance. At a basic level, ionising radiation produces reactive oxygen species which damage DNA. To repair this damage, cells require adequate amounts of raw materials—nucleotides. ‘Switching off’ NRF2 shuts down key metabolic pathways leading to an inability to synthesise nucleotides and proteins necessary to repair cellular damage, plausibly resulting in more effective cell kill from radiation. A permanently ‘switched on’ NRF2 signalling cascade produces the opposite effect (Figure 5). ASNS may be the main mechanism by which the metabolic effect of NRF2 is transduced within the cell, but its role requires further elucidation, which is ongoing within our group.

The inherent novelty of the work herein implies that more elaboration is required before being translated into a clinical setting. As a canonical pathway, which functions as a transcription regulator, it is a possible therapeutic target, with some success already seen in directly inhibiting NRF2 [6,34]. Off target effects are a concern, however, with difficulties targeting NRF2 with direct, specific inhibitors [37]. More recent data have suggested that in order to be active and promote transcription, NRF2 must be de-glycated by Fructosamine-3-kinase (FN3K)—a protein kinase [38]. Sanghvi et al. suggest this may present an opportunity for therapeutic intervention. Another elegant solution may come from exploiting the metabolic shift seen in NRF2 over expressing cancers. A metabolic requirement for glutaminolysis in KEAP1-NRF2-mutant lung adenocarcinoma has been observed, and treatment with two glutaminase inhibitors greatly reduced growth potential [9]. CB839, a glutaminase inhibitor, is currently being used in two phase I/II trials in CRC (NCT03263429; NCT02861300). Interestingly, the former requires KRAS WT patients, while the latter mandates PIK3CA mutant patients. It has been suggested that NRF2 pathway overexpression is a mechanism of acquired tolerance in the PI3K-RAS mutant cancers [27,39] so one may expect the latter study to be enriched for high NRF2 expression and thus more likely to show benefit. Other attractive future strategies could include using an NRF2 biomarker, histological [40] or transcriptomic [25], for enriching studies of metabolic interventions.

## 5. Conclusions

CRC can now be counted among the increasing number of tumours in which NRF2 is a relevant signalling pathway for therapeutic resistance. As its role in metabolic regulation is increasingly understood, translational efforts to exploit the dependency for therapeutic advantage should be encouraged.

## Figures and Tables

**Table 1:** Rectal cohort characteristics (N = 127).

		N	%
Gender	Male	77	61
	Female	50	39
cT stage	1	1	<1
	2	21	17
	3	100	79
	4	5	4
cN	0	63	50
	1	51	40
	2	13	10
ypT	0	33	26
	1	16	13
	2	33	26
	3	45	35
ypN	0	101	80
	1	21	16
	2	5	4
NAR tertiles	Low (0–8)	44	34
	Intermediate (8–16)	56	44
	High (>16)	27	21
Pathological Response *	Complete	33	27
	Incomplete	90	73

* Four patients not formally graded for pathological response.

## Data Availability

Data is contained within the article and supplementary material. RNA sequencing data have been deposited into the Gene Expression Omnibus under accession number GSE136011.

## Supplementary Materials

The supplementary materials are available online at https://www.mdpi.com/article/10.3390/antiox10091380/s1. Figure S1: Additional experimental data including technical replicates, Figure S2: GSEA enrichment plots of significant opposing changes in mTOR sig-nalling, Figure S3: STRING interaction network highlights ASNS at the centre of the reg-ulatory hub, Table S1. NRF2 E79K+ mutant GSEA REACTOME analysis. All significant results (FDR < 0.05) are included. This analysis supports a metabolic upregulation following NRF2 pathway activation, Table S2. NRF2 knockdown GSEA REACTOME analysis. All significant results (FDR < 0.05) are included. This analysis supports a downregulation amino acid and protein metabolism following NRF2 pathway silencing, Table S3. NRF2 E79K+ mutant GSEA Oncogenic signature analysis, Table S4. NRF2 knockdown mutant GSEA Oncogenic signature analysis, Table S5. List of gene names for the 216 overlapping genes from the RNAseq analysis, Table S6. List of 141 gene names that were shared but were differentially expressed in opposing directions, Table S7. List of the 36 genes that constitute the NRF2 expression signature.

## References

[1] Fokas E., Ströbel P., Fietkau R., Ghadimi M., Liersch T., Grabenbauer G.G., Hartmann A., Kaufmann M., Sauer R., Graeven U. (2017). Tumor Regression Grading After Preoperative Chemoradiotherapy as a Prognostic Factor and Individual-Level Surrogate for Disease-Free Survival in Rectal Cancer. JNCI J. Natl. Cancer Inst..

[2] Yoo N.J., Kim H.R., Kim Y.R., An C.H., Lee S.H. (2012). Somatic Mutations of the KEAP1 Gene in Common Solid Cancers. Histopathology.

[3] Hanada N., Takahata T., Zhou Q., Ye X., Sun R., Itoh J., Ishiguro A., Kijima H., Mimura J., Itoh K. (2012). Methylation of the KEAP1 Gene Promoter Region in Human Colorectal Cancer. BMC Cancer.

[4] DeNicola G.M., Karreth F.A., Humpton T.J., Gopinathan A., Wei C., Frese K., Mangal D., Yu K.H., Yeo C.J., Calhoun E.S. (2011). Oncogene-Induced Nrf2 Transcription Promotes ROS Detoxification and Tumorigenesis. Nature.

[5] Sporn M.B., Liby K.T. (2012). NRF2 and Cancer: The Good, the Bad and the Importance of Context. Nat. Rev. Cancer.

[6] Jaramillo M.C., Zhang D.D. (2013). The Emerging Role of the Nrf2–Keap1 Signaling Pathway in Cancer. Genes Dev..

[7] Rojo de la Vega M., Chapman E., Zhang D.D. (2018). NRF2 and the Hallmarks of Cancer. Cancer Cell.

[8] Singh A., Bodas M., Wakabayashi N., Bunz F., Biswal S. (2010). Gain of Nrf2 Function in Non-Small-Cell Lung Cancer Cells Confers Radioresistance. Antioxid. Redox Signal..

[9] Romero R., Sayin V.I., Davidson S.M., Bauer M.R., Singh S.X., LeBoeuf S.E., Karakousi T.R., Ellis D.C., Bhutkar A., Sánchez-Rivera F.J. (2017). *Keap1* Loss Promotes *Kras*-Driven Lung Cancer and Results in Dependence on Glutaminolysis. Nat. Med..

[10] Wang T., Hu P., Li B., Zhang J.-P., Cheng Y.-F., Liang Y.-M. (2017). Role of Nrf2 Signaling Pathway in the Radiation Tolerance of Patients with Head and Neck Squamous Cell Carcinoma: An in Vivo and in Vitro Study. OncoTargets Ther..

[11] Namani A., Rahaman M.M., Chen M., Tang X. (2018). Gene-Expression Signature Regulated by the KEAP1-NRF2-CUL3 Axis Is Associated with a Poor Prognosis in Head and Neck Squamous Cell Cancer. BMC Cancer.

[12] (2017). The Cancer Genome Atlas Research Network Integrated Genomic Characterization of Oesophageal Carcinoma. Nature.

[13] Chio I.I.C., Jafarnejad S.M., Ponz-Sarvise M., Park Y., Rivera K., Palm W., Wilson J., Sangar V., Hao Y., Öhlund D. (2016). NRF2 Promotes Tumor Maintenance by Modulating MRNA Translation in Pancreatic Cancer. Cell.

[14] Sekhar K.R., Freeman M.L. (2015). Nrf2 Promotes Survival Following Exposure to Ionizing Radiation. Free Radic. Biol. Med..

[15] Gonzalez-Donquiles C., Alonso-Molero J., Fernandez-Villa T., Vilorio-Marqués L., Molina A.J., Martín V. (2017). The NRF2 Transcription Factor Plays a Dual Role in Colorectal Cancer: A Systematic Review. PLoS ONE.

[16] Network T.C.G.A. (2012). Comprehensive Molecular Characterization of Human Colon and Rectal Cancer. Nature.

[17] Levings D.C., Wang X., Kohlhase D., Bell D.A., Slattery M. (2018). A Distinct Class of Antioxidant Response Elements Is Consistently Activated in Tumors with NRF2 Mutations. Redox Biol..

[18] Braselmann H., Michna A., Heß J., Unger K. (2015). CFAssay: Statistical Analysis of the Colony Formation Assay. Radiat. Oncol..

[19] Ritz C., Baty F., Streibig J.C., Gerhard D. (2015). Dose-Response Analysis Using R. PLoS ONE.

[20] Love M.I., Huber W., Anders S. (2014). Moderated Estimation of Fold Change and Dispersion for RNA-Seq Data with DESeq2. Genome. Biol..

[21] Robinson M.D., McCarthy D.J., Smyth G.K. (2010). EdgeR: A Bioconductor Package for Differential Expression Analysis of Digital Gene Expression Data. Bioinformatics.

[22] Subramanian A., Tamayo P., Mootha V.K., Mukherjee S., Ebert B.L., Gillette M.A., Paulovich A., Pomeroy S.L., Golub T.R., Lander E.S. (2005). Gene Set Enrichment Analysis: A Knowledge-Based Approach for Interpreting Genome-Wide Expression Profiles. Proc. Natl. Acad. Sci. USA.

[23] Liberzon A., Subramanian A., Pinchback R., Thorvaldsdóttir H., Tamayo P., Mesirov J.P. (2011). Molecular Signatures Database (MSigDB) 3.0. Bioinformatics.

[24] Sergushichev A. (2016). An Algorithm for Fast Preranked Gene Set Enrichment Analysis Using Cumulative Statistic Calculation. bioRxiv.

[25] O’Cathail S.M., Wu C.-H., Lewis A., Holmes C., Hawkins M.A., Maughan T. (2020). NRF2 Metagene Signature Is a Novel Prognostic Biomarker in Colorectal Cancer. Cancer Genet..

[26] Malhotra D., Portales-Casamar E., Singh A., Srivastava S., Arenillas D., Happel C., Shyr C., Wakabayashi N., Kensler T.W., Wasserman W.W. (2010). Global Mapping of Binding Sites for Nrf2 Identifies Novel Targets in Cell Survival Response through ChIP-Seq Profiling and Network Analysis. Nucleic Acids Res..

[27] Mitsuishi Y., Taguchi K., Kawatani Y., Shibata T., Nukiwa T., Aburatani H., Yamamoto M., Motohashi H. (2012). Nrf2 Redirects Glucose and Glutamine into Anabolic Pathways in Metabolic Reprogramming. Cancer Cell.

[28] Spitzner M., Roesler B., Bielfeld C., Emons G., Gaedcke J., Wolff H.A., Rave-Fränk M., Kramer F., Beissbarth T., Kitz J. (2014). STAT3 Inhibition Sensitizes Colorectal Cancer to Chemoradiotherapy in Vitro and in Vivo. Int. J. Cancer.

[29] Krall A.S., Xu S., Graeber T.G., Braas D., Christofk H.R. (2016). Asparagine Promotes Cancer Cell Proliferation through Use as an Amino Acid Exchange Factor. Nat. Commun..

[30] Szklarczyk D., Morris J.H., Cook H., Kuhn M., Wyder S., Simonovic M., Santos A., Doncheva N.T., Roth A., Bork P. (2017). The STRING Database in 2017: Quality-Controlled Protein–Protein Association Networks, Made Broadly Accessible. Nucleic Acids Res..

[31] George T.J., Allegra C.J., Yothers G. (2015). Neoadjuvant Rectal (NAR) Score: A New Surrogate Endpoint in Rectal Cancer Clinical Trials. Curr. Colorectal Cancer Rep..

[32] Fokas E., Fietkau R., Hartmann A., Hohenberger W., Grützmann R., Ghadimi M., Liersch T., Ströbel P., Grabenbauer G.G., Graeven U. (2018). Neoadjuvant Rectal Score as Individual-Level Surrogate for Disease-Free Survival in Rectal Cancer in the CAO/ARO/AIO-04 Randomized Phase III Trial. Ann. Oncol..

[33] Kang K.A., Piao M.J., Ryu Y.S., Kang H.K., Chang W.Y., Keum Y.S., Hyun J.W. (2016). Interaction of DNA Demethylase and Histone Methyltransferase Upregulates Nrf2 in 5-Fluorouracil-Resistant Colon Cancer Cells. Oncotarget.

[34] Evans J.P., Winiarski B.K., Sutton P.A., Jones R.P., Ressel L., Duckworth C.A., Pritchard M.D., Lin Z.-X., Vicky F.L., Tweedle E.M. (2018). The Nrf2 Inhibitor Brusatol Is a Potent Antitumour Agent in an Orthotopic Mouse Model of Colorectal Cancer. Oncotarget.

[35] Wang X.J., Li Y., Luo L., Wang H., Chi Z., Xin A., Li X., Wu J., Tang X. (2014). Oxaliplatin Activates the Keap1/Nrf2 Antioxidant System Conferring Protection against the Cytotoxicity of Anticancer Drugs. Free Radic. Biol. Med..

[36] DeNicola G.M., Chen P.-H., Mullarky E., Sudderth J.A., Hu Z., Wu D., Tang H., Xie Y., Asara J.M., Huffman K.E. (2015). NRF2 Regulates Serine Biosynthesis in Non-Small Cell Lung Cancer. Nat. Genet..

[37] Menegon S., Columbano A., Giordano S. (2016). The Dual Roles of NRF2 in Cancer. Trends Mol. Med..

[38] Sanghvi V.R., Leibold J., Mina M., Mohan P., Berishaj M., Li Z., Miele M.M., Lailler N., Zhao C., de Stanchina E. (2019). The Oncogenic Action of NRF2 Depends on De-Glycation by Fructosamine-3-Kinase. Cell.

[39] Sanchez-Vega F., Mina M., Armenia J., Chatila W.K., Luna A., La K.C., Dimitriadoy S., Liu D.L., Kantheti H.S., Saghafinia S. (2018). Oncogenic Signaling Pathways in The Cancer Genome Atlas. Cell.

[40] Torrente L., Maan G., Oumkaltoum Rezig A., Quinn J., Jackson A., Grilli A., Casares L., Zhang Y., Kulesskiy E., Saarela J. (2020). High NRF2 Levels Correlate with Poor Prognosis in Colorectal Cancer Patients and with Sensitivity to the Kinase Inhibitor AT9283 In Vitro. Biomolecules.

