# Supplementary material for: NRF2 Mediates Therapeutic Resistance to Chemoradiation in Colorectal Cancer through a Metabolic Switch
Source: Antioxidants (Basel). 2021 Aug 28;10(9):1380. doi: 10.3390/antiox10091380 (PMC8466195; doi:10.3390/antiox10091380)
Supplement: Supplementary file 1 [file antioxidants-10-01380-s001.zip › Supplementary methods_Antioxidants_revised.pdf]

Supplementary methods

Cell lines

SW146, HT55 and HCT116 cell lines were provided by Prof Ian Tomlinson (University of Birmingham). Isogenic cell lines $HCT116^{E79K}$ and parental $HCT116^{WT}$ were obtained from Horizon Discovery®. $HCT116^{E79K}$ contains a CRISPR Cas9 engineered *NFE2L2* missense point mutation c.235 G>A. All cells were maintained in Dulbecco's modified Eagles Medium (DMEM) supplemented with 10% FBS and 1% penicillin streptomycin. All cultures were maintained at 37 °C in a humidified 5% CO2 atmosphere and tested to ensure absence of Mycoplasma contamination. Cells were washed with PBS, incubated with trypsin and re-suspended in fresh medium. Cell concentration was determined with Countess™ Cell Counting System (Invitrogen®) as per the manufacturer's instructions. Cell counts were done in duplicate and an average reading taken. Appropriate cell concentrations were made by diluting with DMEM.

siRNA reagents and conditions

siRNAs were supplied pre-designed as dry powder stock at 5 nmoles (Ambion®). Targeted siRNAs were obtained for NFE2L2, as well as non-targeting (NT1) for negative control and polo-like kinase 1 (PLK1) used as positive control. Visual inspection of cell death caused by PLK1 transfection was confirmed by trypan blue staining before proceeding, with cell death rate >50% sufficient. The siRNA sequences are summarised in the table below.

siNFE2L2_1	5′ GAAUGGUCCUAAAACACCAtt
siNFE2L2_2	5′ CGUUUGUAGAUGACAAUGtt
siNFE2L2_3	5′ CAGUCUUCAUUGCUACUAAtt
siPLK1	5′ GCAAUUACAUGAGCGAGCAtt

siRNAs were transfected into HCT116, SW1463 (final concentration 1.8 μM) using Lipofectamine 2000 (Invitrogen®) and HT55 using INTERFERin-HTS (VWR) (final concentration 20nM) in 6 well plates. Statistical comparison of means was performed using Welch's t-test. Gene expression was demonstrated through the measurement of quantitative reverse transcriptase PCR (qRT-PCR). RNA was extracted using the Qiagen® RNeasy Mini Kit as per the manufacturer's instructions. RNA concentration yields were estimated using the nd-1000 NanoDrop® spectrophotometer. Extracted RNA was converted to cDNA. cDNA was amplified using the High Capacity cDNA reverse transcriptase kit (Applied Biosystems™) as per the manufacturers' instructions.

Gene Expression analysis

Gene expression, following siRNA treatment, was demonstrated through the measurement of quantitative reverse transcriptase PCR. RNA was extracted using the Qiagen® RNeasy Mini Kit as per the manufacturer's instructions. RNA concentration yields were made using the nd-1000 NanoDrop® spectrophotometer. Extracted RNA was converted to cDNA in a two-step PCR process. RNA was diluted with RNA-free water to a final volume of 10 μL and mixed with 1 μL of DNAse 1endonuclease and 1 μL of Mg^{2+}. The final solution was heated to 37 °C for thirty minutes. The reaction was terminated by adding 1 μL of EDTA solution and heated to 65 °C for ten minutes. cDNA was amplified using the High Capacity cDNA reverse transcriptase kit (Applied Biosystems™) as per the manufacturer's instructions.
96 well plates were loaded with triplicate wells for each experimental condition. Quantitative reverse transcriptase PCR of *NFE2L2*, *NQO -1, HMOX-1, TXN and KEAP1* was performed using assay-on-demand primers and probe sets from Applied Biosystems and the ABI 7000 Taqman system (Applied Biosystems). Quantification of RNA gene expression was carried out as using the $\Delta\Delta$ C_t method [1]. The result is termed relative fold change and is expressed graphically as mean ± s.d. Statistical comparison of means is performed using Welch's t-test.

Significance levels are indicated as previous (**P*<0.05, ***P*<0.01, ****P*<0.001 and **** *P*<0.0001).

Clonogenic assays

siRNA transfected cells were plated at appropriate dilutions, irradiated at 2, 4 and 6Gy using a caesium-137 irradiator (Gamma Service GSR D1) and incubated for 14–25 days for colony formation. Colonies were fixed in a solution of acetic acid and methanol 1:3 (v/v) and stained with 0.5% (w/v) crystal violet. A colony was defined to consist of 50 cells or greater. Colonies were counted digitally using GELCOUNT™ (Oxford Optronix) software. Experimental results were confirmed with two technical replicates. Table 2 summarises final plating numbers of cells seeded per well. Statistical analysis and plotting of clonogenic survival curves is performed using the 'CFAssay'package in R [2].

Cell line	0 Gy	2 Gy	4 Gy	6 Gy	Incubation time
SW1463	200	400	800	2000	14
HT55	200	1000	2000	5000	25
HCT116	200	400	800	2000	14
HCT116 (E79K+)	200	400	800	2000	14

Linear Quadratic Modelling

For clonogenic assays involving cell irradiation, the experimental data were fitted with the linear quadratic model (LQ):

$S = \exp^{-(\alpha D+\beta D2)}$

where,

S is the survival probability
D the radiation dose (Gy)
α and β are the fitted parameters (Gy^{-1} and Gy^{-2} respectively).

The sensitisation enhancement ratio (SER) was used to quantify radiosensitization (the SER_{10} was deduced from data by using $SER_{10} = D_{control}/ D_{treated}$, where $D_{control}$ and $D_{treated}$ doses yield 10% survival for controls and treated cells, respectively).

Statistical Analysis

Statistical analysis and plotting of clonogenic survival curves is performed using the 'CFAssay'package in R [2]. The software adopts a maximum likelihood based approach to the logarithmic survival fractions of the linear quadratic (LQ) curve, which is preferable to the least squares methods. The survival curves from two sets of treatment conditions are compared using an Analysis of Variance (ANOVA) for two model fits. Statistical significance is set at the 0.05 level, two way analysis. All assay data are representative of three independent experiments and are presented as mean ± SEM. from triplicate wells, unless otherwise stated (*P<0.05, **P<0.01 and ***P<0.001, ****P<0.00001).

Cell viability assay

Cytotoxic drugs were obtained from Sigma (oxaliplatin), Flurochem (5-flurouracil) and Sellekchem (SN-38). All cytotoxic drugs were initially dissolved for a stock solution of 2mM. DMSO was used to dissolve 5-FU and SN-38 and sterile water, warmed to 25 °C was used to dissolve oxaliplatin.

The optimum plating density for all cell lines used was 5000 cell per well in a 96 well plate. An 8 point 3.16 fold dilution of each drug was performed in a 96 well plate (concentration range 2 mM to 636 nM). Cells were plated 24 hours prior to treatment into a 96 well plate. The following day 0.4 μL of drug was added to the 6 replicate wells from the stock plate. The final drug concentration ranges from 8μM to 2.5 nM, with a final DMSO concentration of 0.4%. At 72 hours, the MTS assay (CellTiter® 96R AQueous Non-Radioactive Cell Proliferation Assay) is performed as per the manufacturer's instruction. In brief, 20 μL of the

solution is added to each well and returned to the incubator for 2 hours. The absorbance is read at 490 nM using a photospectrometer. Absorbance shows a direct 1:1 correlation with cell viability.

Statistical Analysis of cell viability assay

Background absorbance from the GM wells is averaged and subtracted from each well. Absorbance in the DMSO well was used to assess the viability of cells in the absence of drug. Absorbance of each well is calculated as a percentage of the absorbance in the DMSO well only. Percentage absorption is a direct surrogate for cell viability. Results are imported in to R for analysis with 'drc package'[3]. Dose-response curves are generated by fitting a four parameter log-logistic regression model, where the independent variable is concentration of drug whilst the dependent variable is the effect of cell viability. Plots indicate cell viability on the y-axis and the log of drug concentration of the x-axis. Effective concentration for 50% cell viability (EC_{50}) is calculated. Statistical significance is calculated by analysis of variance (ANOVA) between the regression models for both sets of conditions, where $p < 0.05$ is considered statistically significant.

Cell line karyotyping

Fluorescent in situ hybridization (FISH) was carried out in collaboration the structural genomics group at the Wellcome Trust Centre for Human Genetics. The following Bacterial Artificial Chromosomes (BAC) and plasmids were used for hybridisation and identification of the NFE2L2 locus [**Error! Reference source not found.**].

Gene locus and loci of the flanking probes used in FISH analysis assess for cytogenetic abnormality at the gene locus

**BAC clone**	**Colour**
pBSD4 2 alphasat: chromosome 2 specific centromeric repeat	Green
RP11-157E8 chr2:177,343,817 -177,497,286	Red

**NFE2L2** chr2: 178,095,031-178,129,859	
RP11-65L3 chr2:179,258,141-179,430,959	Orange
RP11-463B12 chr2:240,911,276 -241,082,695	Purple

RP11-157E8 and RP11-65L3 flank *NFE2L2*, marking the opposite boundaries of the cytogenetic band of where the gene is located (2q31). Upon DNA condensation, the two signals should almost overlap on metaphase chromosomes. If the two flanking BACs are always present together, one can deduce that the chromosomes were not rearranged and so infer that the NFE2L2 gene was also present. Thus, each combined RP11-157E8 and RP11-65L3 signal correspond to one copy of NFE2L2.

All of the probes were hybridised to metaphase spreads from the cell lines, and a minimum of 20 metaphases were analysed per line. As a control for the FISH experiment, the BACs were labelled and hybridised to metaphase spreads from a control cell line. All of the probes mapped to the correct genomic location in the control cells. Results were expressed as a ratio of the number of signals observed per cell.

Sanger sequencing

Sanger sequencing was carried out to confirm the heterozygote mutation of isogenic HCT line using the following primers: F primer GCGACGGAAAGAGTATGAGC; R primer GGAGGCTGAGGTTGGAAAGT.

RNA sequencing

Isogenic HCT116^{WT} and HCT116^{E79K+} mutant cell lines and siRNA treated SW1463 were used for next generation RNA sequencing. RNA was extracted using the Qiagen® RNeasy Mini Kit as per the manufacturer's instructions quantified using the nd-1000 NanoDrop® spectrophotometer. DNase treated RNA was checked for concentration and purity prior to

submission for RNA sequencing. All samples with a RNA Integrity Number (RIN) of 7 or higher were processed to generate libraries for RNA sequencing following the Illumina TruSeq stranded RNA sample preparation guide. Poly-A enriched mRNA libraries were sequenced as a 12 sample multiplex on one lane of an Illumina HiSeq4000 machine, performing 75bp paired end sequencing. Approximately 20 million reads were obtained per sample. Reads were aligned to the human reference genome (GRCh37) using HISAT2 [4] and duplicate reads removed using the Picard 'MarkDuplicates' tool (http://broadinstitute.github.io/picard/). Reads mapping uniquely to Ensembl-annotated genes (~15 million per sample) were summarised using featureCounts [5]. The raw gene count matrix was imported into the R/BioConductor environment for further processing and analysis.

Differential expression

Differential expression analysis was performed with both 'DESeq2' [6] and 'edgeR' [7] to ensure robustness. Main analysis and plots were generated from edgeR data. Genes were initially filtered out with read counts of <1 count per million (cpm) in at least 4 samples. Normalisation factors for the library sizes were determined in using the trimmed mean method (TMM) in edgeR. Relative to the control, gene dispersion of the perturbed system was estimated before fitting to a negative binomial model. Genes with a FDR of =< 0.01 were selected to check for overlap with DESeq2 as an estimation of agreement between the two packages.

Gene Set Enrichment Analysis

GSEA was carried out against the hallmark gene sets from the Molecular Signatures Database v6.2 [8, 9] (http://software.broadinstitute.org/gsea/downloads.jsp) using the 'fgsea' package[10]. The ranked gene list was compared to the *a priori* defined gene sets in the hallmarks genesets and the curated geneset NFE2L2.v2, for a total of 1000 permutations. A

FDR <0. 1 was used to highlight significant pathways of enrichment. Venn diagrams of overlap were created using www.interactivenn.com.

Rectal cohort

The dataset comprised of rectal biopsy samples sequentially collected from patients that have been treated with radiotherapy/ chemoradiotherapy. Access to this dataset, including the RNA expression profiles, was through the MRC Stratification in Colorectal cancer (S:CORT) consortium. All samples were annotated by a single pathologist for pathological tumour (ypT) and pathological nodal stage (ypN) as well as pathological regression grade (complete, good partial, partial and minimal). Reference H&E slides were marked for areas of adenocarcinoma in the rectal biopsies and adjacent sections were cut for multi-omics profiling.

Sample handling and RNA extraction

The work was carried out by multiple members of the S:CORT collaboration and provided for context. Stored tissue blocks were transferred to Leeds University sample processing where 1 x 5 micron and 2 x 10 micron sections were cut. The 5 micron slide was H&E stained, annotated for the tumour region and all sections were shipped to Queen's University Belfast for processing. Samples were dewaxed by an automated process using the Tissue-Tek Prisma machine, using a combination of xylene washes and ethanol. Macrodissections were performed within a designated macrodissection area treated with RNase decontamination solution. The unstained section was overlaid with the annotated H&E slide and using a clean scalpel blade the annotated area was scraped into a 1.5ml RNase-free Eppendorf tube containing the prepared Roche High Pure RNA Paraffin Kit tissue lysis reagent (catalogue 0327089001).

RNA extraction as per the Roche High Pure RNA Paraffin Kit instructions. Extracted RNA was quantified by Nanodrop and stored at -80 C. Samples which had sufficient RNA concentration were submitted for profiling by cRNA and ds-cDNA preparation before being subjected to further quality control. Finally samples were hybridised to the Xcel microarray as per the manufacturer's instructions. RNA profiles were submitted to Oxford as raw CEL files. Quality control analysis was run on samples using the R base 'AffyQC' module (https://github.com/BiGCAT-UM/affyQCModule). The files were then processed using the R packages 'limma' (https://bioconductor.org.packages/release/bioc/html/limma.html) and 'affy' (https://bioconductor.org.packages/release/bioc/html/affy.html) to normalise the expression values using robust multiarray algorithm (RMA) and generate and expression matrix of probe intensities against the samples. All samples were batch corrected for outcome variables including date of scan.

Construction of the NRF2 signature

As this work is described elsewhere in the referenced paper [11], in brief, the NRF2 signature was constructed by performing PCA on the corresponding probe sets matched from the training set. Major PCs were identified using the same filtering method for the training set, where PCs are unsupervised summary statistics of the probe expressions. The genes which make up the 36 gene signature are *ABCA8, ABI3BP, ADAM12, ADRB1, ANGPT1, ANKRD29, ANKRD44, BCHE, C15orf48, COL3A1, COL5A1, EGLN3, LIFR, METTL7A, PCM1, PLAU, PLCB4, RECK, RGCC, RRM2, SEC14L4, SERPINH1, SFN, SLIT3, SPP1, TNS1, TOM1L2, TSPAN5, TTYH3, VSIG10, VCAN, AKR1C1, LRP8, NAMPT, PTGES, SLC27A5*. This group of genes was designated NRF2 signature going forward.

Statistical analysis

The primary analysis was to test whether the NRF2 signature explained the NAR score. An ordinal logistic regression model was constructed without any variables to represent the null model. Another ordinal logistic regression model was built with the NRF2 signature. The null hypothesis (H0) was that NRF2 would not provide any explanatory power for NAR score. The likelihood ratio test (LRT) was employed to inform the strength of evidence against the null hypothesis. To investigate whether the NRF2 signature was confounded with other known variables that (potentially) explained radiotherapy response, an adjusted analysis was performed using a multivariate ordinal logistic regression model. An ordinal logistic regression model was constructed with clinical nodal stage (cN). Another ordinal logistic regression models was constructed with the NRF2 metagene and clinical nodal stage (cN). A subsequent LRT was performed to test the null hypothesis that the model with the NRF2 signature did not add to the explanatory power of the model. Statistical significance from the LRT indicated that there was evidence for the NRF2 signature providing additional information to explain the NAR.

1. Livak, K.J. and T.D. Schmittgen, *Analysis of relative gene expression data using real-time quantitative PCR and the 2(-Delta Delta C(T)) Method.* Methods, 2001. **25**(4): p. 402-8.
2. Braselmann, H., et al., *CFAssay: statistical analysis of the colony formation assay.* Radiat Oncol, 2015. **10**: p. 223.
3. Ritz, C., et al., *Dose-Response Analysis Using R.* PLoS One, 2015. **10**(12): p. e0146021.
4. Kim, D., B. Langmead, and S.L. Salzberg, *HISAT: a fast spliced aligner with low memory requirements.* Nat Methods, 2015. **12**(4): p. 357-60.
5. Liao, Y., G.K. Smyth, and W. Shi, *featureCounts: an efficient general purpose program for assigning sequence reads to genomic features.* Bioinformatics, 2014. **30**(7): p. 923-30.
6. Love, M.I., W. Huber, and S. Anders, *Moderated estimation of fold change and dispersion for RNA-seq data with DESeq2.* Genome Biol, 2014. **15**(12): p. 550.
7. Robinson, M.D., D.J. McCarthy, and G.K. Smyth, *edgeR: a Bioconductor package for differential expression analysis of digital gene expression data.* Bioinformatics, 2010. **26**(1): p. 139-40.

8. Liberzon, A., et al., *Molecular signatures database (MSigDB) 3.0.* Bioinformatics, 2011. **27**(12): p. 1739-40.
9. Subramanian, A., et al., *Gene set enrichment analysis: a knowledge-based approach for interpreting genome-wide expression profiles.* Proc Natl Acad Sci U S A, 2005. **102**(43): p. 15545-50.
10. Korotkevich G, S.V., Sergushichev A *Fast gene set enrichment analysis.* bioRxiv, 2019.
11. O'Cathail, S.M., et al., *NRF2 metagene signature is a novel prognostic biomarker in colorectal cancer.* Cancer Genet, 2020. **248-249**: p. 1-10.
