# Supplementary material for: NRF2 Mediates Therapeutic Resistance to Chemoradiation in Colorectal Cancer through a Metabolic Switch
Source: Antioxidants (Basel). 2021 Aug 28;10(9):1380. doi: 10.3390/antiox10091380 (PMC8466195; doi:10.3390/antiox10091380)
Supplement: Supplementary file 1 [file antioxidants-10-01380-s001.zip › Supplementary Tables.pdf]

Supplementary Table S1. NRF2 E79K+ mutant GSEA REACTOME analysis. All significant results (FDR<0.05) are included. This analysis supports a metabolic upregulation following NRF2 pathway activation.

NAME	SIZE	ES	NES	NOM p-val	FDR q-val	FWER p-val
REACTOME_CYTOSOLIC_TRNA_AMINOACYLATION	24	0.799234	2.365734	0	0.00115	0.001
REACTOME_AMINO_ACID_TRANSPORT_ACROSS_THE_PLASMA_MEMBRANE	19	0.751057	2.093708	0	0.034043	0.059
REACTOME_3_UTR_MEDIATED_TRANSLATIONAL_REGULATION	101	0.541479	2.085299	0	0.026658	0.07
REACTOME_PEPTIDE_CHAIN_ELONGATION	83	0.548106	2.082995	0	0.021336	0.075
REACTOME_TRNA_AMINOACYLATION	42	0.62209	2.054241	0.0025	0.023814	0.1
REACTOME_TRANSLATION	140	0.504411	2.053282	0	0.020044	0.1
REACTOME_ACTIVATION_OF_GENES_BY_ATF4	21	0.712194	2.051942	0	0.017493	0.102
REACTOME_SRP_DEPENDENT_COTRANSLATIONAL_PROTEIN_TARGETING_TO_MEMBRANE	106	0.516262	2.023135	0	0.019919	0.13
REACTOME_NONSENSE_MEDIATED_DECAY_ENHANCED_BY_THE_EXON_JUNCTION_COMPLEX	103	0.497637	1.951462	0	0.034997	0.249
REACTOME_AMINO_ACID_AND_OLIGOPEPTIDE_SLC_TRANSPORTERS	28	0.636472	1.951289	0.0023	0.031497	0.249
REACTOME_CLASS_A1_RHODOPSIN_LIKE_RECEPTORS	48	-0.67473	-2.14845	0	0.001957	0.002

Supplementary Table S2. NRF2 knockdown GSEA REACTOME analysis. All significant results (FDR<0.05) are included. This analysis supports a downregulation amino acid and protein metabolism following NRF2 pathway silencing.

NAME	SIZE	ES	NES	NOM p-val	FDR q-val	FWER p-val
REACTOME_PEPTIDE_CHAIN_ELONGATION	83	-0.5631	-2.27579	0	0	0
REACTOME_AMINO_ACID_SYNTHESIS_AND_INTERCONVERSION_ TRANSAMINATION	16	-0.76231	-2.10528	0	0.0033	0.011
REACTOME_3_UTR_MEDIATED_TRANSLATIONAL_REGULATION	101	-0.49432	-2.04397	0	0.0046	0.023
REACTOME_SRP_DEPENDENT_COTRANSLATIONAL_PROTEIN_ TARGETING_TO_MEMBRANE	106	-0.4896	-2.01264	0	0.0048	0.032
REACTOME_METABOLISM_OF_AMINO_ACIDS_AND_DERIVATIVES	147	-0.44709	-1.94989	0	0.0085	0.068
REACTOME_INFLUENZA_VIRAL_RNA_TRANSCRIPTION_AND_REPLICATION	99	-0.47132	-1.92226	0	0.0106	0.097
REACTOME_TIGHT_JUNCTION_INTERACTIONS	17	-0.67322	-1.92183	0	0.0091	0.098
REACTOME_FORMATION_OF_THE_TERNARY_COMPLEX_AND_ SUBSEQUENTLY_THE_43S_COMPLEX	47	-0.52488	-1.88128	0	0.0135	0.163
REACTOME_TRANSLATION	140	-0.43641	-1.87026	0	0.0130	0.175
REACTOME_NONSENSE_MEDIATED_DECAY_ENHANCED_BY_THE_EXON_ JUNCTION_COMPLEX	103	-0.44695	-1.85767	0	0.0136	0.2
REACTOME_CYTOSOLIC_TRNA_AMINOACYLATION	24	-0.61101	-1.84534	0	0.0142	0.227
REACTOME_BIOLOGICAL_OXIDATIONS	57	-0.49328	-1.82073	0	0.0173	0.289
REACTOME_PHASE1_FUNCTIONALIZATION_OF_COMPOUNDS	24	-0.57366	-1.78451	0	0.0239	0.397
REACTOME_CELL_CELL_JUNCTION_ORGANIZATION	28	-0.54238	-1.7345	0.0095	0.0374	0.573
REACTOME_ACTIVATION_OF_THE_MRNA_UPON_BINDING_OF_ THE_CAP_BINDING_COMPLEX_AND_EIFS_AND_SUBSEQUENT_BINDING_TO_43S	54	-0.46797	-1.72003	0	0.0399	0.618
REACTOME_CELL_JUNCTION_ORGANIZATION	46	-0.47985	-1.70153	0	0.0447	0.679
REACTOME_ACTIVATION_OF_THE_PRE_REPLICATIVE_COMPLEX	30	0.796686	2.612971	0	0	0
REACTOME_G2_M_CHECKPOINTS	41	0.729244	2.557482	0	0	0
REACTOME_DNA_REPLICATION	182	0.561845	2.540232	0	0	0
REACTOME_DNA_STRAND_ELONGATION	30	0.789028	2.5142	0	0	0
REACTOME_ACTIVATION_OF_ATR_IN_RESPONSE_TO_REPLICATION_STRESS	35	0.739281	2.508276	0	0	0
REACTOME_MITOTIC_M_M_G1_PHASES	163	0.552593	2.453981	0	0	0
REACTOME_G1_S_TRANSITION	105	0.594157	2.4522	0	0	0
REACTOME_CELL_CYCLE_MITOTIC	297	0.509199	2.441214	0	0	0
REACTOME_E2F_MEDIATED_REGULATION_OF_DNA_REPLICATION	32	0.742291	2.425769	0	0	0
REACTOME_MITOTIC_G1_G1_S_PHASES	128	0.564756	2.417765	0	0	0
REACTOME_CELL_CYCLE	349	0.49407	2.379414	0	0	0
REACTOME_S_PHASE	104	0.564118	2.34609	0	0	0
REACTOME_G1_S_SPECIFIC_TRANSCRIPTION	16	0.834893	2.328178	0	0	0
REACTOME_M_G1_TRANSITION	77	0.585096	2.323481	0	0	0
REACTOME_SYNTHESIS_OF_DNA	88	0.561304	2.28107	0	0	0

REACTOME_CHROMOSOME_MAINTENANCE	78	0.571357	2.267552	0	0	0
REACTOME_MITOTIC_PROMETAPHASE	82	0.560776	2.250004	0	7.93E-05	0.001
REACTOME_EXTENSION_OF_TELOMERES	27	0.703272	2.226929	0	7.49E-05	0.001
REACTOME_LAGGING_STRAND_SYNTHESIS	19	0.737602	2.211822	0	7.10E-05	0.001
REACTOME_G0_AND_EARLY_G1	22	0.715282	2.14326	0	4.09E-04	0.006
REACTOME_TELOMERE_MAINTENANCE	42	0.608501	2.129606	0	5.30E-04	0.008
REACTOME_CELL_CYCLE_CHECKPOINTS	110	0.488123	2.026893	0	0.0018	0.028
REACTOME_GLOBAL_GENOMIC_NER_GG_NER	32	0.606256	2.0155	0	0.0021	0.034
REACTOME_HOMOLOGOUS_RECOMBINATION_REPAIR_OF_ REPLICATION_INDEPENDENT_DOUBLE_STRAND_BREAKS	16	0.702464	1.955537	0	0.0046	0.075
REACTOME_MEIOTIC_RECOMBINATION	30	0.588501	1.899733	0	0.0094	0.152
REACTOME_DEPOSITION_OF_NEW_CENPA_CONTAINING_ NUCLEOSOMES_AT_THE_CENTROMERE	29	0.598102	1.897923	0.0020	0.0091	0.152
REACTOME_PROCESSIVE_SYNTHESIS_ON_THE_LAGGING_STRAND	15	0.706243	1.89348	0.0020	0.0093	0.16
REACTOME_DOUBLE_STRAND_BREAK_REPAIR	22	0.639913	1.891811	0.002	0.0093	0.167
REACTOME_ASSEMBLY_OF_THE_PRE_REPLICATIVE_COMPLEX	62	0.48884	1.840454	0	0.0154	0.262
REACTOME_MEIOSIS	52	0.509952	1.835602	0.0020	0.0157	0.275
REACTOME_MITOTIC_G2_G2_M_PHASES	73	0.474167	1.833079	0.0020	0.0156	0.281
REACTOME_TRANSPORT_OF_MATURE_TRANSCRIPT_TO_CYTOPLASM	52	0.499501	1.818727	0	0.0179	0.326
REACTOME_FANCONI_ANEMIA_PATHWAY	18	0.606267	1.762154	0.0021	0.0328	0.535
REACTOME_DNA_REPAIR	100	0.423156	1.755409	0	0.0337	0.558
REACTOME_G_ALPHA_I_SIGNALLING_EVENTS	44	0.496325	1.748535	0	0.0347	0.576

Supplementary Table S3. NRF2 E79K+ mutant GSEA Oncogenic signature analysis.

NAME	SIZE	ES	NES	NOM p-val	FDR q-val	FWER p-val
ALK_DN.V1_UP	52	0.670215	2.299545	0	0	0
**MTOR_UP.N4.V1_UP**	**165**	**0.469496**	**1.967683**	**0**	**0.006403**	**0.019**
KRAS.PROSTATE_UP.V1_DN	51	-0.66015	-2.14918	0	0	0
MYC_UP.V1_DN	108	-0.56792	-2.05149	0	5.19E-04	0.001
CAHOY_ASTROGLIAL	59	-0.60038	-1.98144	0	0.001844	0.005
VEGF_A_UP.V1_UP	118	-0.53079	-1.93401	0	0.003465	0.012
KRAS.300_UP.V1_DN	51	-0.59428	-1.89346	0	0.004441	0.019
PTEN_DN.V1_UP	68	-0.54459	-1.84096	0	0.008824	0.044
KRAS.50_UP.V1_UP	17	-0.71142	-1.83732	0.006838	0.007724	0.045
KRAS.600_UP.V1_UP	109	-0.50326	-1.81759	0	0.0091	0.061
E2F3_UP.V1_DN	95	-0.50628	-1.7997	0	0.010246	0.076
NRL_DN.V1_DN	89	-0.50452	-1.79742	0	0.009565	0.079
CSR_LATE_UP.V1_DN	120	-0.48118	-1.78774	0	0.009232	0.084
LEF1_UP.V1_UP	127	-0.48234	-1.77498	0	0.009843	0.097
KRAS.300_UP.V1_UP	55	-0.541	-1.74953	0	0.012793	0.133
ESC_V6.5_UP_EARLY.V1_DN	116	-0.47018	-1.72378	0	0.016253	0.176
MEK_UP.V1_UP	164	-0.44416	-1.70286	0	0.01981	0.224
KRAS.DF.V1_UP	148	-0.45098	-1.69347	0.001468	0.020375	0.243
KRAS.600_UP.V1_DN	94	-0.46443	-1.68108	0	0.022356	0.277
WNT_UP.V1_UP	92	-0.46474	-1.65521	0	0.028167	0.347
PTEN_DN.V2_DN	100	-0.46704	-1.65345	0.003049	0.02705	0.351
ESC_J1_UP_EARLY.V1_UP	126	-0.44211	-1.64985	0.002994	0.026521	0.361
PRC2_EZH2_UP.V1_UP	121	-0.44413	-1.63661	0.001515	0.029221	0.41
ATF2_S_UP.V1_DN	108	-0.45064	-1.63398	0.001522	0.02901	0.417
KRAS.LUNG.BREAST_UP.V1_DN	51	-0.51224	-1.62768	0.013029	0.029498	0.437
ATM_DN.V1_DN	53	-0.51461	-1.62572	0.006472	0.028806	0.445
TGFB_UP.V1_DN	138	-0.4367	-1.61358	0.00146	0.032095	0.498

YAP1_DN	29	-0.5665	-1.60421	0.014286	0.033546	0.523
AKT_UP.V1_DN	116	-0.4399	-1.59714	0.003077	0.034952	0.547
E2F3_UP.V1_UP	151	-0.41642	-1.58301	0.004511	0.039827	0.617
ALK_DN.V1_DN	59	-0.47934	-1.57656	0.015437	0.04124	0.643
BMI1_DN.V1_UP	91	-0.44349	-1.56748	0.00753	0.043838	0.68
ERB2_UP.V1_UP	152	-0.41685	-1.56326	0.004418	0.044392	0.697
BCAT_BILD_ET_AL_UP	40	-0.49852	-1.56048	0.021812	0.044442	0.711
PTEN_DN.V1_DN	76	-0.45739	-1.55768	0.01129	0.044231	0.718

Supplementary Table S4. NRF2 knockdown mutant GSEA Oncogenic signature analysis.

NAME	SIZE	ES	NES	NOM p-val	FDR q-val	FWER p-val
IL21_UP.V1_UP	92	-0.47337	-1.90405	0	0.012854	0.011
**MTOR_UP.N4.V1_UP**	**171**	**-0.39813**	**-1.77579**	**0**	**0.036614**	**0.059**
ALK_DN.V1_UP	56	-0.47246	-1.75122	0.004049	0.030048	0.072
PTEN_DN.V1_UP	69	-0.45327	-1.73777	0.001862	0.026064	0.084
AKT_UP.V1_DN	117	-0.41141	-1.71746	0	0.02664	0.108
SIRNA_EIF4GI_UP	81	-0.43633	-1.71697	0	0.0222	0.108
LEF1_UP.V1_DN	130	-0.4017	-1.7097	0	0.020873	0.117
RB_P107_DN.V1_DN	110	-0.39854	-1.6641	0.001818	0.030548	0.194
STK33_SKM_DN	163	-0.37701	-1.66388	0	0.027154	0.194
NFE2L2.V2	248	-0.35701	-1.6618	0	0.025256	0.199
SINGH_KRAS_DEPENDENCY_SIGNATURE_	16	-0.59216	-1.65568	0.023576	0.025196	0.213
CRX_DN.V1_UP	81	-0.40831	-1.64652	0.003906	0.02516	0.233
STK33_DN	180	-0.35853	-1.62032	0.001908	0.031088	0.294
KRAS.600.LUNG.BREAST_UP.V1_DN	116	-0.38068	-1.58068	0.005882	0.04256	0.398
P53_DN.V1_UP	151	-0.35903	-1.57641	0.001946	0.041446	0.411
ESC_V6.5_UP_LATE.V1_DN	133	-0.36218	-1.56657	0.002033	0.043682	0.448
MEL18_DN.V1_DN	80	-0.40281	-1.56446	0.007921	0.042061	0.455
STK33_NOMO_DN	175	-0.34682	-1.54792	0	0.047221	0.525
RB_DN.V1_DN	98	-0.37802	-1.5444	0.009579	0.046309	0.538
KRAS.50_UP.V1_UP	19	-0.54168	-1.53935	0.039698	0.04613	0.554
EIF4E_UP	72	-0.39256	-1.51047	0.008032	0.058685	0.651
RB_P107_DN.V1_UP	108	0.553455	2.293732	0	0	0
CSR_LATE_UP.V1_UP	142	0.519952	2.268417	0	0	0
GCNP_SHH_UP_LATE.V1_UP	154	0.465391	2.054174	0	0	0
E2F3_UP.V1_UP	135	0.476593	2.047229	0	0	0
PRC2_EED_UP.V1_DN	166	0.434526	1.927086	0	0.001655	0.01
E2F1_UP.V1_UP	163	0.429576	1.90736	0	0.001526	0.011
VEGF_A_UP.V1_DN	160	0.41161	1.841662	0	0.002444	0.021
RPS14_DN.V1_DN	160	0.413383	1.809671	0	0.003578	0.035
SRC_UP.V1_DN	114	0.411419	1.737402	0	0.007485	0.078
CORDENONSI_YAP_CONSERVED_SIGNATURE	45	0.475667	1.704668	0	0.010228	0.117
ERB2_UP.V1_DN	161	0.367355	1.655628	0	0.016828	0.201
GCNP_SHH_UP_EARLY.V1_UP	140	0.370996	1.614719	0.002028	0.024544	0.301
PRC2_EZH2_UP.V1_DN	127	0.363001	1.571153	0.002165	0.035774	0.444
CAHOY_NEURONAL	33	0.474663	1.5679	0.023061	0.034399	0.455
HOXA9_DN.V1_DN	152	0.356651	1.567665	0	0.032106	0.455
STK33_NOMO_UP	199	0.339778	1.540373	0.002151	0.039461	0.549
PTEN_DN.V2_DN	97	0.373564	1.525151	0.008282	0.043874	0.606
CAMP_UP.V1_DN	156	0.340292	1.515154	0	0.045645	0.647

Table S5. List of gene names for the 216 overlapping genes from the RNAseq analysis

Gene name
AARS
ABCC2
ABLIM1
ACSL3
ACTN4
ADGRB2
ADGRG1
ADM
AK2
ALDOC
ANGPTL4
ANXA1
ANXA2
AREG
ARHGAP23
ARHGAP29
ARL4C
ARRB1
ASAH1
ASNS
ASS1
ATF4
ATL3
BCL3
BHLHE40
BMP4
BTN3A2
BUD31
CALCOCO2
CAMK2D
CAPG
CARS
CDH1
CDK1
CEBPG
CHAC1
CIC
CLDN7
COQ5
CRABP2
CTSB
CYBRD1
CYR61
DDIT4
DEPDC1
DHCR24
DHRS2
DIXDC1

DNMT3B
DPYSL3
EHBP1
EHF
EIF2S2
EIF4EBP1
ELF3
EMP3
EPB41L1
EPRS
ERMP1
ESRP1
ETV4
FAM111B
FAM83D
FAM84B
FDXR
FGFBP1
FHL2
FLOT1
FTL
FUS
GARS
GATA2
GDA
GDF15
GLO1
GNAI1
GPT2
GRB10
GSN
GTF3C4
H1F0
HAS3
HAX1
HEATR5A
HKDC1
HLA-E
HNF4A
HOXB5
HOXB6
HOXB8
HSPA8
HYOU1
ID1
IER3
IFI6
IGFBP3
INADL
INSIG1
ITGA3

ITGB4
JADE2
KIF18A
KLF5
KLK6
L3MBTL3
LAD1
LAMA3
LAMC2
LGALS1
LPCAT1
LPCAT3
LRP11
LRRC45
LSS
MAGI1
MAP1B
MAP7
MARS
MBNL1
MCC
MET
MTHFD2
MYL9
NARS
NDRG1
NIPSNAP1
NRAS
NT5E
NTSR1
NUP155
NUP160
OBFC1
PALLD
PCK2
PCTP
PGPEP1
PHGDH
PHTF2
PIK3R3
PLCB3
PRKCDBP
PRRC2B
PRSS33
PSAT1
PSME1
PSPH
PTPN1
PTPN14
PTRF
PYCR1

PYGB
RAD18
RASSF8
RBCK1
RNF145
RSL24D1
RUNX1
SAMD9
SARS
SAT1
SCG2
SDCBP
SEL1L3
SEMA3C
SEMA4B
SERPINB6
SERPINE1
SERPINH1
SESN2
SESN3
SGOL2
SH3BP4
SH3KBP1
SHMT2
SKI
SLC16A5
SLC1A4
SLC1A5
SLC2A6
SLC3A2
SLC43A1
SLC4A5
SLC7A5
SMARCA1
SNN
SNTB1
SOX9
SREBF1
SRSF6
ST14
STC2
SYDE1
TAPBP
TBCD
TBX3
TES
TGFBR3
TMEM2
TRIB3
TRIP13
TSEN15

TSPAN5
TUBE1
TXNRD1
UBE2H
UBTF
ULBP1
UNC5B
UPP1
WARS
XPOT
YARS
YOD1
ZCCHC24
ZNF367
ZNF503

Table S6. List of 141 gene names that were shared but were differentially expressed inopposing directions.

Gene name
AARS
ABLIM1
ACSL3
ACTN4
ADGRB2
ADGRG1
ADM
AK2
ANXA1
ANXA2
AREG
ARHGAP23
ARL4C
ARRB1
ASNS
ASS1
ATF4
ATL3
BMP4
BUD31
CALCOCO2
CAMK2D
CARS
CDH1
CEBPG
CHAC1
CIC
CLDN7
COQ5
CTSB
CYBRD1
DDIT4

DHCR24
DIXDC1
DPYSL3
EHBP1
EIF2S2
EIF4EBP1
EPRS
ESRP1
ETV4
FAM111B
FAM84B
FGFBP1
FHL2
FTL
GARS
GATA2
GDA
GDF15
GNAI1
GPT2
GRB10
GTF3C4
H1F0
HAX1
HEATR5A
HKDC1
HOXB5
HOXB6
HOXB8
HYOU1
IER3
IFI6
INADL
INSIG1
JADE2
KIF18A
KLK6
LAD1
LAMA3
LAMC2
LGALS1
LPCAT1
LPCAT3
LRRC45
LSS
MAP1B
MAP7
MARS
MBNL1
MET
MTHFD2

MYL9
NARS
NIPSNAP1
NT5E
NTSR1
PCK2
PCTP
PGPEP1
PHGDH
PIK3R3
PLCB3
PSAT1
PSPH
PTRF
PYCR1
PYGB
RSL24D1
SARS
SAT1
SCG2
SEL1L3
SEMA3C
SERPINE1
SESN2
SESN3
SH3KBP1
SHMT2
SKI
SLC1A4
SLC1A5
SLC2A6
SLC3A2
SLC43A1
SLC4A5
SLC7A5
SMARCA1
SNN
SOX9
SREBF1
ST14
STC2
SYDE1
TBCD
TBX3
TES
TRIB3
TSEN15
TUBE1
TXNRD1
UBTF
ULBP1

UPP1
WARS
XPOT
YARS
ZCCHC24
ZNF367
ZNF503

Table S7. List of the 36 genes that constitute the NRF2 expression signature.

Gene name
ABCA8
ABI3BP
ADAM12
ADRB1
ANGPT1
ANKRD29
ANKRD44
BCHE
C15orf48
COL3A1
COL5A1
EGLN3
LIFR
METTL7A
PCM1
PLAU
PLCB4
RECK
RGCC
RRM2
SEC14L4
SERPINH1
SFN
SLIT3
SPP1
TNS1
TOM1L2
TSPAN5
TTYH3
VSIG10
VCAN
AKR1C1
LRP8
NAMPT
PTGES
SLC27A5
