# Supplementary figures and images for: NRF2 Mediates Therapeutic Resistance to Chemoradiation in Colorectal Cancer through a Metabolic Switch
Source: Antioxidants (Basel). 2021 Aug 28;10(9):1380. doi: 10.3390/antiox10091380 (PMC8466195; doi:10.3390/antiox10091380)

## Slide 1
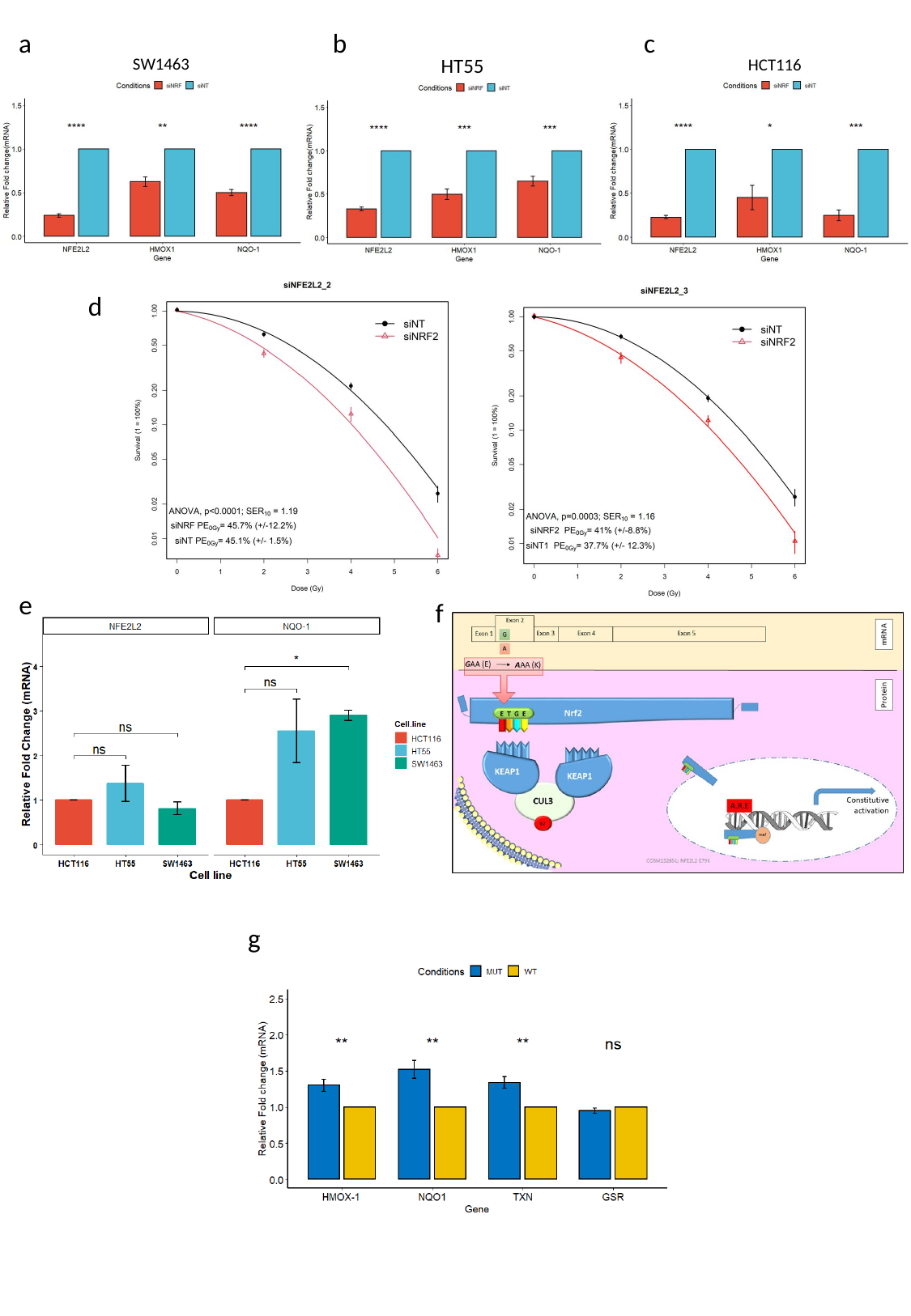

a
b
c
HT55
SW1463
HCT116
d
e
f
g

Supplement: Supplementary file 1 [file antioxidants-10-01380-s001.zip › Supp Figure S1.pptx]

## Slide 1
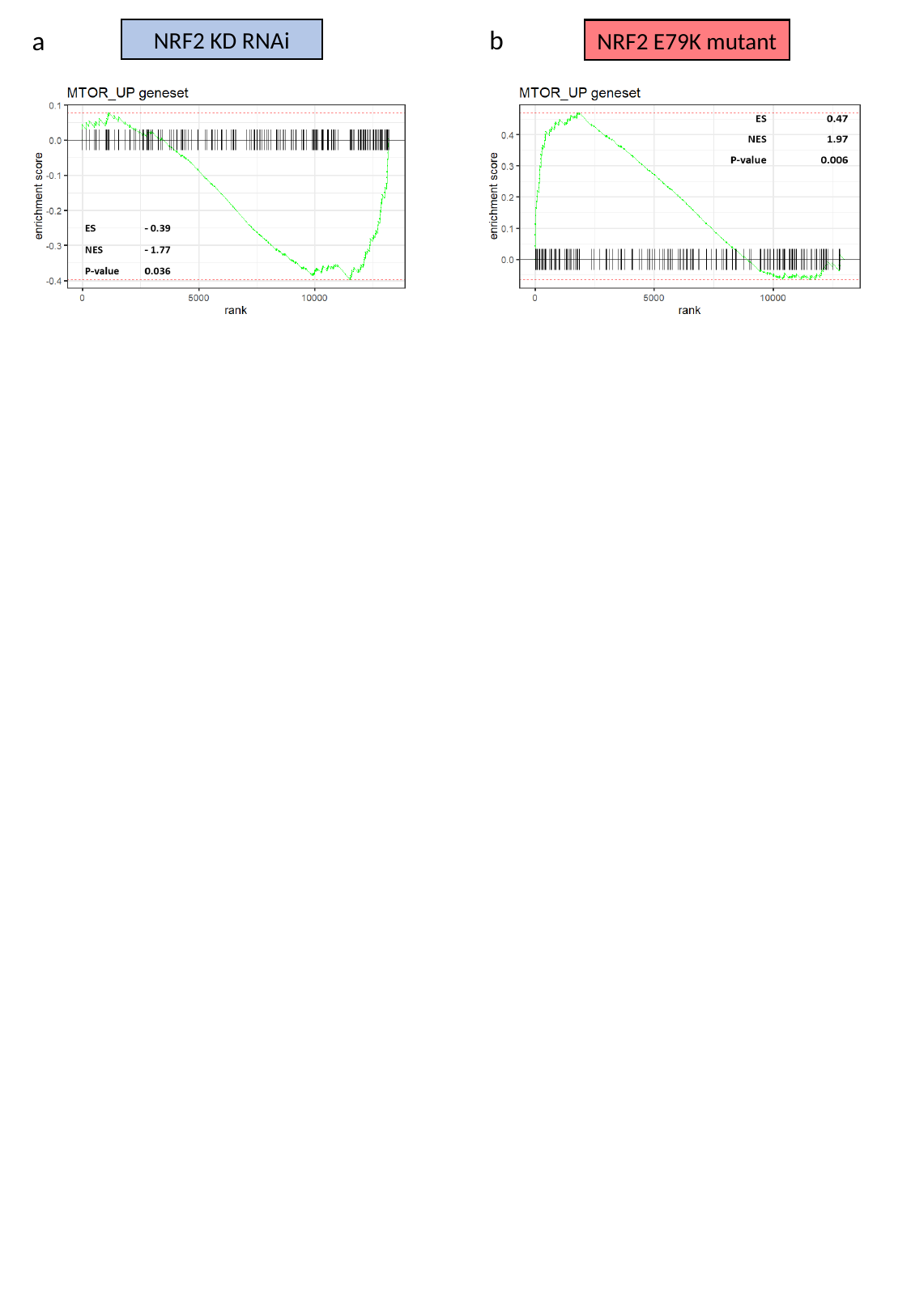

NRF2 KD RNAi
NRF2 E79K mutant
b
a

Supplement: Supplementary file 1 [file antioxidants-10-01380-s001.zip › Supplementary Figure S2.pptx]

## Slide 1
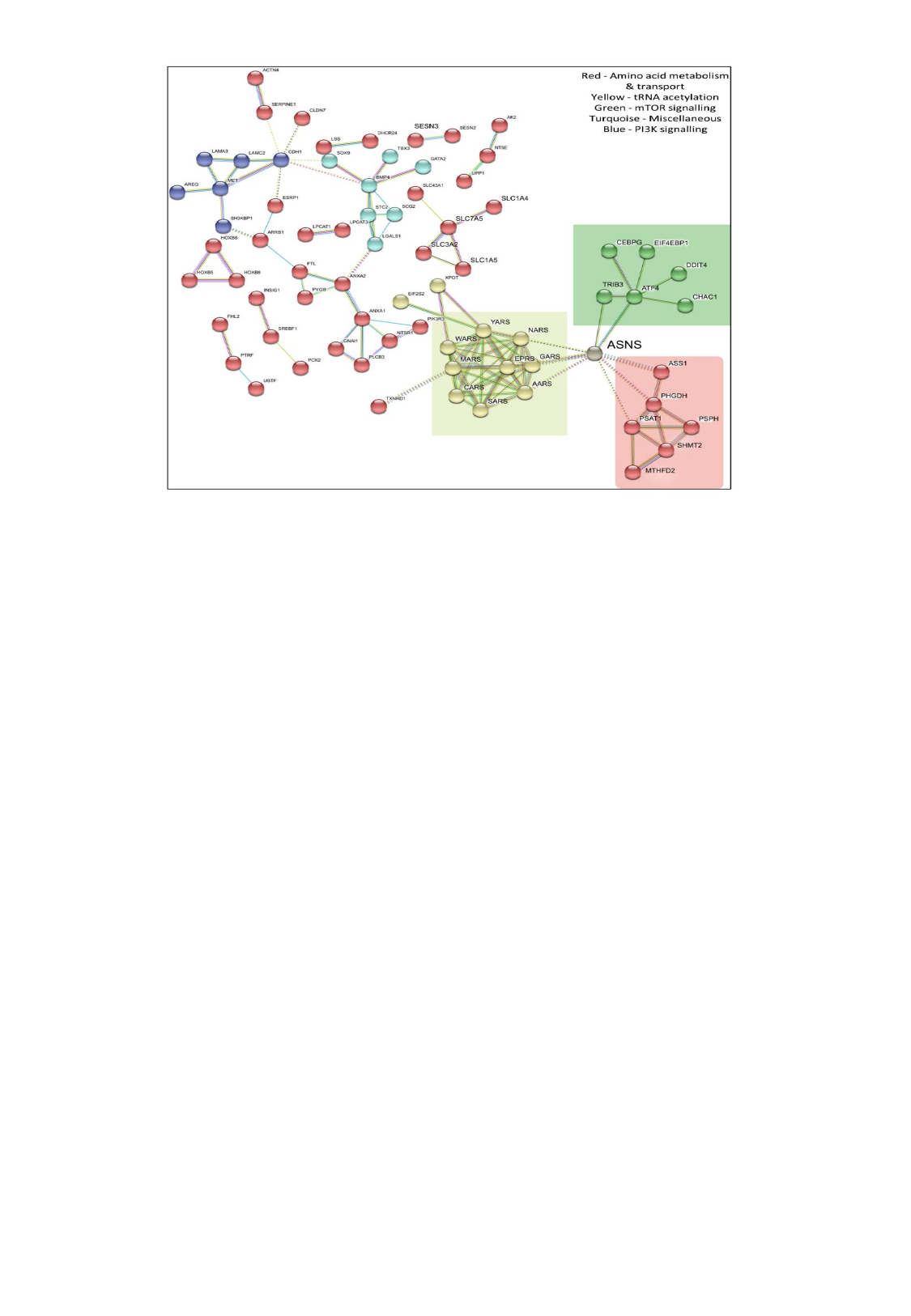

Supplement: Supplementary file 1 [file antioxidants-10-01380-s001.zip › Supplementary Figure S3.pptx]
